# Robot-assisted transvesical simple prostatectomy with circumferential mucosal anastomosis: long-term urinary and sexual function outcomes in a 292 patient cohort

**DOI:** 10.1007/s11701-025-02879-0

**Published:** 2025-10-16

**Authors:** Luke Shumaker, Zev Leopold, Max Perilstein, Samuel Ricci, Daniel Eun

**Affiliations:** Fox Chase-Temple Urologic Institute, Philadelphia, PA USA

**Keywords:** Benign prostatic hyperplasia (BPH), Obstructive uropathy, Robotic surgery, Simple prostatectomy, Prostate adenoma

## Abstract

To assess the long-term urinary and sexual function outcomes for men undergoing robotic-assisted simple prostatectomy (RASP) with circumferential mucosal anastomosis performed at a single, high-volume robotics center. RASP cases performed by a single surgeon from June 2013 through June 2024 using the da Vinci^®^ Xi robotic system were analyzed. Indications for surgery were bothersome lower urinary tract symptoms (LUTS) refractory to medical management and prostate volume ≥ 80 g. Urinary function and sexual function parameters were assessed by the American Urologic Association Symptoms Score (AUASS) and Sexual Health Inventory for Men (SHIM), respectively at routine 6-month postoperative visits. A uniform phone survey was conducted in September 2024, which assessed long-term functional outcomes by combining AUASS with questions on incontinence, erectile function, and orgasm. A Wilcoxon signed rank test was utilized to compare pre- and postoperative IPSS and SHIM scores with *p* < 0.05 considered significant. 292 patients underwent RASP during the query interval and were included in the perioperative analysis with a mean follow-up time of 22.6 months (SD ± 15.2). Mean preoperative AUASS was 17.9 (SD ± 7.9). Mean postoperative AUASS was 5.7 (SD ± 5.2); *W*-statistic 352.0 (*p* < 0.001). Bother scores preoperative mean was 4.3 (SD ± 1.5), with postoperative values of 1.1 ± 1.4; *W*-statistic 395.0 (*p* < 0.001). Sexual Health Inventory for Men (SHIM) scores demonstrated a preoperative mean of 12.8 (SD ± 8.1). Postoperatively, the SHIM mean was 12.6 (SD ± 8.5); *W*-statistic: 4462.0 (*p* = 0.57). No patients required reoperation for LUTS, although one patient underwent completion prostatectomy for malignancy identified on RASP pathology. No patients developed bladder neck contracture. Calls were placed to 288 patients (98.6% of total cohort) at a mean follow up of 66 (SD ± 34.7) months postoperatively, of which 198 (68.8%) answered and consented to survey participation. At the time of the follow-up survey, mean IPSS was 2.1 (SD ± 1.9), mean quality of life score was 0.7 (SD ± 0.8). With respect to continence, 196/198 (98.9%) patients experienced no stress incontinence. Two patients (1%) experienced urge incontinence and one patient (0.5%) utilized an incontinence pad. Regarding sexual activity, 157 (79.3%) patients were sexually active at the time of survey compared to 159 (80.3%) prior to surgery. Of those who remained sexually active, 152 (96.8%) were satisfied with postoperative orgasm, and 149 (94.9%) were satisfied with postoperative erectile function. Notably, 11 (7.0%) of men endorsed a “bothersome or distressing” orgasm change. RASP has a low complication rate, low risk of urinary incontinence, no significant impact on erectile function, and provides durable improvements in lower urinary tract symptoms. A small portion of men do experience sustained, bothersome orgasm change following RASP.

## Introduction

Benign prostatic hyperplasia (BPH) is a common condition in aging men, characterized by the non-malignant enlargement of the prostate gland, leading to obstructing and irritative lower urinary tract symptoms (LUTS). As BPH progresses, it can lead to significant impact on quality of life due to urinary retention, frequency, urgency, nocturia and catheter-dependance. While medical therapy with alpha-blockers or 5-alpha reductase inhibitors is first-line treatment, options for LUTS, surgical intervention after failed medical therapy is common.

For the large prostate glands, those greater than 80 grams, the American Urologic Association (AUA) recommends simple prostatectomy or laser enucleation of the prostate (LEP) [1]. Historically, simple prostatectomy was performed by an open approach, termed open simple prostatectomy (OSP). The first robotic-assisted simple prostatectomy was reported in 2008 and the majority of simple prostatectomies are now performed using a robotic-assisted laparoscopic approach [2]. In 2013, our institution began a novel modification to RASP by performing the adenectomy through a posterior cystotomy incision followed by a complete 360-degree anastomosis of the bladder neck to the prostatic urethra, thereby effectively sequestering the raw, post-adenectomy dead space. Preliminary outcomes for this technique suggested it was safe and effective [3]. Simple prostatectomy has also been performed using single-port robotic systems with promising outcomes [4]. RASP offers several advantages over open prostatectomy, including shorter hospital stays, faster recovery, and less blood loss [5–7]. In addition to these perioperative benefits, the functional outcomes, particularly concerning urinary and sexual health, remain critical considerations in the management of BPH. Short and intermediate-term urinary outcomes after RASP are generally favorable, with marked improvement in LUTS, even in those with a history of prior endoscopic intervention for BPH [8, 9]. Similarly, while RASP leads to anejaculation, studies have shown minimal rates of postoperative erectile dysfunction [9, 10]. More recently, some surgeons have sought to modify RASP to preserve ejaculation [11, 12]. Independent of technique or modification, there is little published data on long-term functional outcomes after RASP.

We sought to analyze the sexual and urinary function outcomes in our institutional RASP cohort using routinely collected SHIM and AUASS data in addition to a uniformly administered phone survey with the goal of quantifying long-term outcomes.

## Materials and methods

### Patient cohort

We conducted a retrospective review of all patients who underwent RASP with circumferential mucosal anastomosis from June 2013 through June 2024. All procedures were performed by a single surgeon using the da Vinci^®^ Xi robotic surgical system. The indication for intervention was catheter dependent urinary retention or bothersome LUTS in patients with gland size greater than 80 g as calculated by preoperative transrectal ultrasonography or axial pelvic imaging. All patients underwent preoperative cystourethroscopy to evaluate prostate anatomy and assess the bladder for tumors or other unexpected pathology. Patients that demonstrated one or more medium or large bladder diverticula routinely underwent concurrent diverticulectomy at the time of RASP.

### Surgical technique

As previously described by our institution, we modified the traditional anterior, retropubic approach by accessing the adenectomy via a posterior transvesical entry with extirpation of the adenoma followed by a circumferential 360-degree running vesicourethral mucosal anastomosis [13].

### Access

Using a 0-degree scope on a multiport robotic system (daVinci Si or Xi surgical system, Sunnyvale, CA), transperitoneal access is made using four 8 mm robotic ports and 2 assistant ports (5 and 12 mm), similar to the port schema used for radical prostatectomy. A midline cystotomy incision is created from the dome to the supratrigonal bladder, posteriorly. The bladder is not mobilized, and retraction sutures are not necessary for the bladder wall. This approach allows for direct visualization of the prostatic fossa and bladder trigone without violating the space of Retzius, dorsal venous complex, the sphincter, or the endopelvic fascia.

### Adenectomy

A u-shaped incision is opened at mucosa under the median lobe and to access the posterior adenoma plane between the peripheral zone and the transition zone. The location of the ureteral orifices are noted carefully and avoided. It is typical to encounter bleeding from the posterior bladder neck mucosa. Once this plane is opened and bladder neck bleeding controlled with cautery, blunt dissection along the adenoma plane is followed as deeply towards the apex as possible. If the vas, seminal vesicles or neurovascular bundle planes are encountered, the dissection plane needs to be re-oriented away from the capsular plane and back to the adenoma plane. Once the posterior adenoma has been freed, this dissection plane is followed laterally while opening the bladder neck mucosa as tightly as possible, which will ultimately help make the mucosa anastomosis easier after adenectomy. Laterally, the space should be taken as deep to the apex as possible while ensuring that the capsular plane has not been entered. Anteriorly, as the bladder neck has completely been disconnected from the adenoma, it is imperative that the dissection plane clearly define the borders of the anterior commissure, effectively demonstrating a “widow’s peak” configuration just above the intra-prostatic urethra. If the dissection is incorrectly made outside of this plane, the dorsal venous complex can be entered and lead to significant bleeding. If the anterior apical dissection is continued at the capsular level, the sphincter complex is at risk of unnecessary dissection, thereby leading to the possibility of continence issues after surgery. A proper dissection anteriorly should outline the sulcus that defines the anterior commissure. When the anterior commissure is defined, this plane is used to guide the distal aspect the adenoma dissection while sparing the verumontanum with all the critical distal structures including the external urethral sphincter. If the adenoma is very bulky and distal dissection is too deep to enable reliable visualization, the anterior commissure can be divided to remove a prostate hemi-lobe. This allows the adenoma to be taken in parts as necessary to allow for good visualization during the distal dissection. After the adenoma is removed and bagged, careful hemostasis within the fossa is achieved using cautery and/or interrupted sutured.

### 360-degree anastomosis and cystotomy closure

Conventionally, following removal of the adenoma, the prostatic fossa is left open to the bladder. Hemostasis is achieved by a combination of catheter traction and bladder irrigation as needed. In contrast, we circumferentially advance the bladder mucosa and muscular bladder neck to the distal prostatic urethra using a running 12-inch absorbable 3–0 barbed suture on a CV-23 needle (VLOC™, Medtronic, Minneapolis, MN). This creates a complete mucosa-to-mucosa anastomosis, effectively excluding urine from mixing with potential bleeding from the prostatic fossa. After the anastomosis is complete, a thrombin based hemostatic matrix (Floseal^®^, Baxter International, Deerfield IL), is injected via sterile arterial line tubing into the prostatic fossa dead space. Finally, the posterior cystotomy is closed in two, water-tight layers using running 3–0 absorbable barbed suture. A two-way 18 French Foley catheter is placed and a leak test performed. A surgical drain is not placed.

### Postoperative care

Patients are typically discharged on the day of surgery from the post-anesthesia recovery unit or on postoperative day one. Continuous bladder irrigation and catheter traction are not used. Patients are discharged with bladder antispasmodics, ibuprofen, and stool softeners. The Foley catheter is maintained for approximately 5 to 7 days. No imaging is obtained prior to catheter removal.

### AUASS, SHIM, and phone survey data

**Fig. 1:**
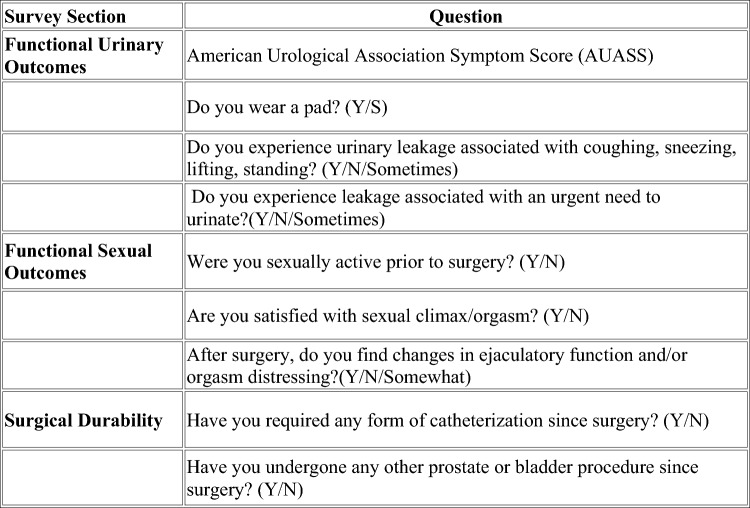
Depicts details of telephonic survey administered to patient in September 2024

Functional survey assessments including AUASS and SHIM were obtained preoperatively as well as postoperatively at 3-, 6-, and 9-month postoperative visits. When multiple surveys were present, the most recent survey result was used. A separate phone survey was conducted in September of 2024, which assessed long-term functional outcomes by combining AUASS with questions directed at incontinence, erectile function, orgasm quality, and need for any additional urologic interventions following RASP. See Fig. 1 for survey details.

### Statistical analysis

Descriptive statistics were calculated for cohort demographics and perioperative data. A Wilcoxon signed rank test was used to compare preoperative and postoperative IPSS and SHIM scores. *p* < 0.05 was considered to be statistically significant. Statistical analyses were conducted via Python and SciPy version 1.15.1.

## Results

Patient demographics and perioperative data are included in Table 1. 292 patients underwent RASP during the designated interval. Mean age was 70.4 years (SD 8.2); and mean BMI was 28.8 kg/m^2^ (SD 4.9). 36 patients (12.33%) were on anticoagulation therapy at the time of their preoperative appointment, 122 patients (41.78%) had prior abdominal surgery, and 31 patients (10.62%) had undergone a prior procedure for BPH. Preoperatively, 282 of the patients (96.52%) experienced lower urinary track symptoms (LUTS), 204 (69.86%) acute urinary retention, 39 (13.36%) gross hematuria, and 32 (10.96%) bladder stones. The mean preoperative prostate volume was 156.1 g (SD 78.7), and 43.6% of patients were dependent on a Foley catheter at the time of surgery.

**Table 1:** Patient demographics, characteristics, and perioperative outcomes (*n* = 292)

Mean age ± SD (years)	70.4 ± 8.2
Mean BMI ± SD (kg/m^2^)	28.8 ± 4.9
History of prior abdominal surgery (%)	41.6%
History of prior prostate surgery (%)	10.5%
Preoperatively Foley catheter dependent (%)	43.6%
Mean preoperative prostate volume ± SD (mL)	156.1 ± 78.7
Mean operative time ± SD (min)	164.1 ± 54.5
Mean estimated blood loss ± SD (mL)	301.8 ± 234.9
Median length of stay [IQR] (days)	1 [0]
Mean duration of postoperative catheter time ± SD (days)	7.3 ± 4.1
Mean clinic follow-up ± SD (months)	22.6 ± 15.2
Complications of Clavien > 2 within 30 days post operatively (%)	2.7%

The mean operative time was 164.1 min (SD 54.5), with a mean estimated blood loss of 301.8 mL (SD 234.9). 252 (85.9%) patients were discharged on POD 0 or POD 1. Additional procedures performed concurrently to RASP included cystolithotomy in 40 patients (13.70%), diverticulectomy in 41 patients (14.04%), and hernia repair in 17 patients (5.82%). There was one intraoperative complication (0.34%), and postoperative complications classified as Clavien–Dindo grade > 2 within 30 days occurred in 8 (2.7%) patients. Postoperative pathology revealed adenocarcinoma in 21/292 (7.2%) patients. The mean postoperative catheter duration was 7.3 days (SD 4.1), and patients had a mean follow-up duration of 22.6 months (SD 15.2).

Figure 1 depicts the assessments of urinary and sexual function as they were evaluated at the preoperative and 6- or 9-month postoperative appointment. A Wilcoxon signed rank test was utilized to compare pre- and postoperative AUASS, bother, and SHIM scores. AUASS had a preoperative mean 17.9 (SD 7.9). Postoperatively, the mean AUASS was 5.7 (SD 5.2), *W*-statistic 352.0 (*p* < 0.001). Bother scores showed a preoperative mean of 4.3 (SD 1.5), with postoperative values of 1.1 (SD 1.4); *W*-statistic 395.0 (*p* < 0.001). SHIM scores demonstrated a preoperative mean of 12.8 ± 8.1. Postoperatively, the SHIM mean was 12.6 ± 8.5; *W*-statistic: 4462.0 (*p* = 0.57).

Survey phone calls were placed to 288 post RASP patients (98.6%). 198 patients (68.8%) answered the survey call and consented to participate. For phone survey participants the mean surgery to survey interval was 66 months (SD 34.7). The mean AUASS was 2.1 (SD 1.9), the mean quality of life score was 0.7 (SD 0.8). With respect to continence, 196/198 (98.9%) patients denied any stress incontinence. 2 (1.0%) experienced urge incontinence; and 1 (0.5%) utilized an incontinence pad. 157 (79.3%) patients were sexually active at the time of survey compared to 159 (80.3%) prior to surgery. Of those who are sexually active, 152 (96.8%) were satisfied with postoperative orgasm, and 149 (94.9%) were satisfied with postoperative erectile function. Notably, 11 (7.0%) of men did endorse a bothersome or distressing orgasm change after RASP.

## Discussion

**Fig. 2:**
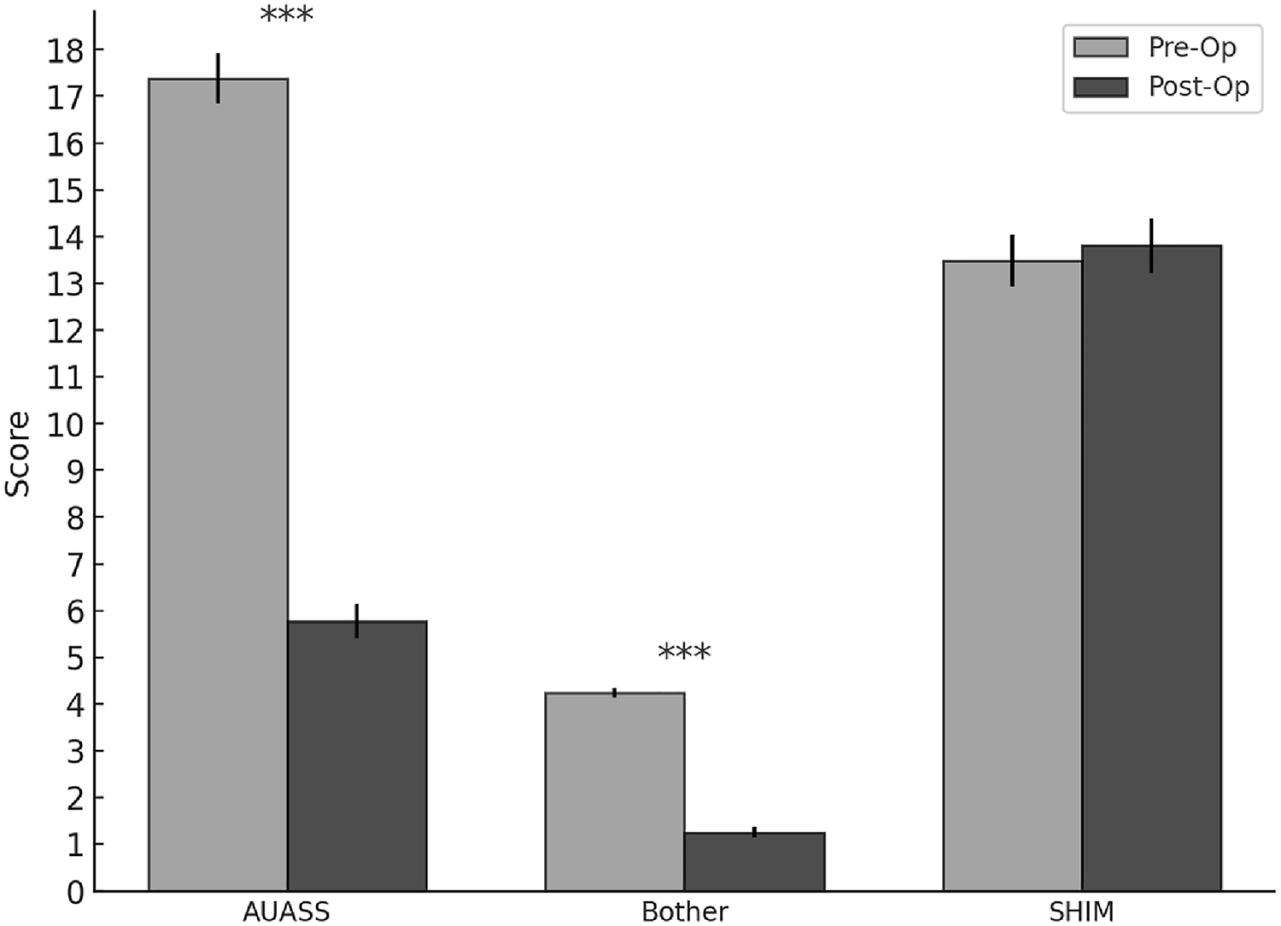
AUASS, Bother score, and SHIM scores at pre and postoperative visits. In this figure, ***indicates (*p* < 0.001)

As RASP grew in popularity for management of large adenomas, it was naturally compared to the previous gold standard of OSP, where it definitively proved to be as effective for relief of LUTS, but with shorter length of stay (LOS), decreased estimated blood loss (EBL), shorter catheter time, and fewer complications [14]. Overall, our study supports those previously published results—namely that RASP is a safe and effective method for treating BPH. Of the 292 RASP patients, 252 (85.9%) were discharged on POD 0 or 1. There was a single, recognized intraoperative complication, a small left distal ureteral injury, which was repaired primarily. Postoperatively, 8 (2.7%) patients experienced a complication of Clavien–Dindo > 2. These included a small bowel obstruction requiring laparoscopic lysis of adhesions, two incisional hernias requiring operative repair, a bladder leak requiring operative repair, an enterotomy requiring reoperation, pulmonary emboli requiring therapeutic anticoagulation, hydronephrosis secondary to pelvic hematoma requiring robotic-assisted hematoma evacuation, and incision site bleeding requiring re-exploration (Fig. 2).

Many prior studies investigating the perioperative and functional outcomes of RASP have compared it to OSP. However, laser enucleation of the prostate (LEP) is now a more relevant comparator. A recent large meta-analysis including six studies and 1235 patients found that while LEP and RASP produced comparable IPSS, Q_max_, PSA, and post-void residual (PVR), LEP offered improved operative time (mean difference (MD)—67.96 min; *p* = 0.04), shorter LOS (MD—2.44 days; *p* < 0.0001), and shorter catheterization time (MD—6.31 days; *p* < 0.0001). Further, LEP accomplished these improvements while providing a lower transfusion rate (OR 0.23, *p* = 0.006) and fewer Clavien–Dindo complications of Grade ≥ 3 (OR 0.435, *p* = 0.049) [15]. Similarly, a retrospective study comparing LEP, OSP, and RASP found that HoLEP was superior to RASP and OSP in terms of operative time, LOS, catheter duration, and EBL [16].

However, a proposed benefit of RASP over LEP is lower rates of urethral stricture or bladder neck contracture (BNC). Prior studies have shown rates of 1.4–4.4% and 0.6 to 5.4% for urethral stricture and BNC following HoLEP [17]. In contrast, in their systematic review Kordan et al. notes BNC rates of < 1% following RASP [18]. In our cohort, we had no observed incidences of bladder neck contracture. We theorize that the circumferential vesicourethral anastomosis aids in prevention of BNC by mucosa-to-mucosa re-approximation of healthy urothelial tissues while sequestering the raw prostatic fossa and reducing the risk of annular, obstructing scar.

Regarding blood loss, our mean ± SD EBL 301.8 mL (SD 234.9). This is in line with previously published perioperative RASP outcomes [14]. Only one patient required re-intervention for persistent bleeding after therapeutic anticoagulation was started on postoperative day 2 for acute pulmonary emboli. No patients required CBI postoperatively. Again, we posit that the circumferential vesicourethral anastomosis technique plays a role in this perioperative outcome by allowing exclusion of the raw prostatic surface, aiding in hemostasis and obviating the need for CBI (Table 2).

**Table 2:** Long-term patient-reported outcomes from postoperative phone survey (*n* = 198)

Mean IPSS ± SD (kg/m^2^)	2.1 ± 1.9
Mean quality of life score ± SD (%)	0.7 ± 0.8
Postoperative stress urinary incontinence (%)	1.4%
Urge incontinence (%)	1%
Use of incontinence pads/briefs (%)	0.5%
Sexually active preoperatively (%)	80.3%
Sexually active at time of survey (%)	79.3%
Satisfied with postoperative erectile function (%)	94.9%
Satisfied with postoperative orgasm (%)	96.8%
Experienced distressful orgasm change (%)	7.0%

In terms of urinary and sexual outcomes, there has yet to be definitive evidence of the superiority of RASP or HoLEP. One study exploring perioperative and mid-term outcomes in 97 patients who underwent holmium laser enucleation of the prostate (HoLEP) or RASP, 56% of patients who underwent RASP were able to achieve *Q*_max_ > 15 mL/s, IPSS < 8, and no complications, compared to only 33% in the LEP group (*p* = 0.02) [19]. Similarly, other studies showed higher rates of transient urinary incontinence in the postoperative period following LEP compared to RASP (8.9 vs. 1.2%; *p* = 0.035) [20]. In our cohort, only 2 patients (1%) noted stress urinary incontinence, and 2 patients (1%) noted mild urge urinary incontinence, comparable to prior published RASP outcomes, and favorable compared to HoLEP [21]. Our group has previously published encouraging intermediate-term functional outcomes at a mean follow-up of 31.3 months (SD 18.2) following RASP [3]. A randomized controlled trial comparing HoLEP to RASP or LSP found that while functional outcomes were generally comparable, a higher percentage of patients who underwent HoLEP experienced new onset irritative LUTS (33.3 vs 13.2%; *p* = 0.02) at one month of follow-up [22]. In addition to the prevention of BNC and its hemostatic benefits, we hypothesize that because the circumferential vesicourethral anastomosis creates continuity of the urothelium, it may improve postoperative irritative symptoms. Most of the current literature investigating functional outcomes following RASP has limited follow-up intervals. To our knowledge, our single-institution study has the longest mean follow-up time of 66 months (SD 34.7) postoperatively. We found that not only does RASP lead to significant improvement in functional urinary outcomes in the perioperative period, but that these improvements are durable over the long term.

Regarding sexual outcomes, our study showed no significant impact on SHIM in the perioperative period. Over the long term, 94.9% were satisfied with postoperative erectile function, 96.8% were satisfied with postoperative orgasm, and 7.0% experienced a distressing or bothersome change in orgasm sensation. This is comparable to prior published HoLEP outcomes, with Roper et al. showing no significant impact on erectile function as measured by SHIM scores (11.51 pre-HoLEP vs. 13.327 post-HoLEP, *p* = 0.498) [23]. However, they did note a significant decline in perceived ejaculatory function as measured by Male Sexual Health Questionnaire—Ejaculatory Dysfunction (8.70 pre-HoLEP vs. 6.58 post HoLEP, *p* ≤ 0.001). Like the aforementioned study, we did appreciate a subgroup of our cohort who were dissatisfied with their postoperative ejaculatory dysfunction. Generally, retrograde/anejaculation was an accepted consequence of simple prostatectomy. However, a recent published by Simone et al. introduced a novel method termed the Madigan technique, which utilizes near-infrared fluorescence imaging to identify and preserve the urethra and ejaculatory ducts in an effort to preserve ejaculatory function [12]. Bove et al. compared this technique to the more traditional trans-vesical (Freyer) or trans-capsular (Millin) method and found that while the Madigan technique had longer operative times, ejaculatory function was preserved in 71% of patients [11]. Further, this translated to favorable sexual outcomes as measured by the International Index of Erectile Function (IIEF) and Male Sexual Health Questionnaire (MSHQ) at one year follow-up. However, individuals in the Madigan cohort were younger and more likely to be sexually active. Nonetheless, this represents a respectable advancement in RASP technique, and a technique worth considering for younger patients who desire preservation of ejaculatory function.

While our study shows excellent, durable functional outcomes following RASP, some limitations should be mentioned. The technique of circumferential vesicourethral anastomosis is not uniformly performed across centers, and thus our outcomes may not be generalizable to RASP outcomes performed without the technique. Furthermore, this was a single arm, retrospective study with no control arm. Therefore, this study by itself does not support an association between the outcomes reported here and the vesicourethral anastomotic technique. Future studies directly comparing our technique with other RASP techniques would help clarify our theorized benefits to restoration of the urothelium over the prostatic fossa. Many prior studies suffer from limited follow-up. We were able to overcome that by contacting prior patients via telephone and administering a survey consisting of the IPSS and selected sexual health questions. Despite the advantages of telephonic surveys, some limitations must be acknowledged. First, selection bias may affect the representativeness of the sample. Individuals without access to a telephone, those with changed contact information, or those who refused to participate in the survey could feasibly have biased survey results. Second, response bias is a concern, as participants may provide socially desirable answers rather than truthful responses, particularly when discussing sensitive topics such as urinary or sexual outcomes in real time via a phone conversation.

## Conclusion

Our study demonstrates that RASP with circumferential vesicourethral anastomosis produces significant improvements in LUTS and minimal impact on erectile function. A small percentage of men do experience sustained bothersome or distressing changes in orgasm following RASP and patients should be counseled regarding this infrequent outcome. To our knowledge, this single-institution experience contains the longest published follow-up to date, signifying that these improvements are safe and durable.

## Data Availability

No datasets were generated or analyzed during the current study.
